# Supplementary material for: Clinical Features of Gastric Signet Ring Cell Cancer: Results from a Systematic Review and Meta-Analysis
Source: Cancers (Basel). 2023 Oct 28;15(21):5191. doi: 10.3390/cancers15215191 (PMC10647446; doi:10.3390/cancers15215191)
Supplement: Supplementary file 1 [file cancers-15-05191-s001.zip › Figure S2.pdf]

Figure S2. Forest plot showing meta-analysis results for survival (comparison of multivariable HR/RR), subgroup analysis by stage and histology (between SRCC and NSRCC, UDC/PDA/mucinous, DC/WMDA/ADK). **(A):** in Early Gastric Cancer patients. **(B):** in Advanced Gastric Cancer patients.

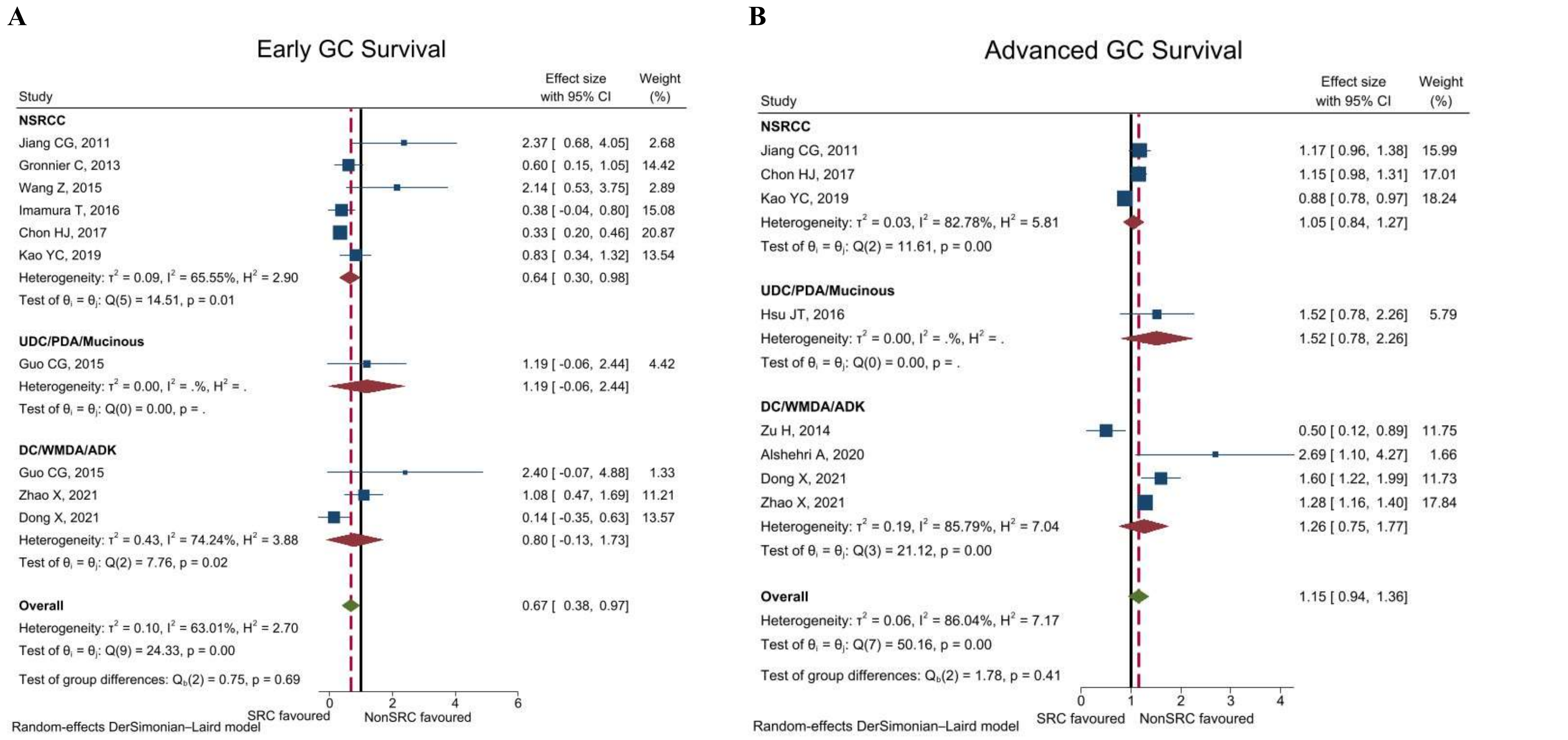

Abbreviations. ADK: Adenocarcinoma not otherwise specified; DC: Differentiated Cancer; GC: Gastric Cancer; NSRCC: Non-Signet Ring Cell Carcinoma; PDA: Poorly Differentiated Adenocarcinoma; SRCC: Signet Ring Cell Carcinoma; WMDA: Well/Moderately Differentiated Adenocarcinoma; UDC: Undifferentiated Cancer.
