# Supplementary material for: Clinical Features of Gastric Signet Ring Cell Cancer: Results from a Systematic Review and Meta-Analysis
Source: Cancers (Basel). 2023 Oct 28;15(21):5191. doi: 10.3390/cancers15215191 (PMC10647446; doi:10.3390/cancers15215191)
Supplement: Supplementary file 1 [file cancers-15-05191-s001.zip › Table S1.pdf]

Table S1. Search terms used in PubMed.

Step	
**1**	((Stomach[MeSH Terms]) OR Stomach[Title/Abstract]) OR Gastric[Title/Abstract] Filters: English; Field: Title/Abstract
**2**	((((((Neoplasm[MeSH Terms]) OR Neoplasm[Title/Abstract]) OR Carcinoma[MeSH Terms]) OR Carcinoma[Title/Abstract]) OR Adenocarcinoma[MeSH Terms]) OR Adenocarcinoma[Title/Abstract]) OR Cancer[Title/Abstract] Filters: English; Field: Title/Abstract
**3**	((((Carcinoma, Signet Ring Cell[Title/Abstract]) OR Carcinoma, Signet Ring Cell[MeSH Terms]) OR Signet Ring[Title/Abstract]) OR Signet Ring Cell[Title/Abstract] Filters: English; Field: Title/Abstract
**4**	Combination 1 AND 2 AND 3
