# Supplementary material for: Clinical Features of Gastric Signet Ring Cell Cancer: Results from a Systematic Review and Meta-Analysis
Source: Cancers (Basel). 2023 Oct 28;15(21):5191. doi: 10.3390/cancers15215191 (PMC10647446; doi:10.3390/cancers15215191)
Supplement: Supplementary file 1 [file cancers-15-05191-s001.zip › Table S2.pdf]

Table S2. Quality assessment of cohort studies according to the Newcastle-Ottawa Scale (NOS).

	Study	Criterion score			Total
		**Selection**	**Comparability**	**Outcome**	
	**Author, year**	★★★★	★★	★★★	**9**
**1**	Efared B 2020	★★★	★	★★	6
**2**	Lee JH 2010	★★★★	★★	★★	8
**3**	Nam MJ 2010	★★★★	★★	★★	8
**4**	Kim HM 2011	★★★	★★	★★	7
**5**	Tong JH 2011	★★★	★★	★★	7
**6**	Huh CW 2013	★★★★	★★	★★	8
**7**	Kim BS 2014	★★★★	★★	★★★	9
**8**	Guo CG 2015	★★★★	★★	★★	8
**9**	Jin EH 2015	★★★★	★★	★★	8
**10**	Lee SH 2015	★★★★	★★	★★	8
**11**	Wang Z 2015	★★★	★★	★★	7
**12**	Hwang CS 2016	★★★	★	★★	6
**13**	Imamura T 2016	★★★	★★	★★	7
**14**	Kim YH 2016	★★★★	★	★	6
**15**	Yoon HJ 2016	★★★★	★	★	6
**16**	Bang CS 2017	★★★	★	★★	6
**17**	Kang Sun H 2017	★★★	★★	★★★	8
**18**	Lee IS 2017	★★★★	★★	★★	8
**19**	Horiuchi Y 2018	★★★★	★★	★★	8
**20**	Kwak DS 2018	★★★★	★★	★★	8
**21**	Nakamura R 2019	★★★	★	★★	6
**22**	Ryu DG 2019	★★★	★★	★★	7
**23**	Zhu ZL 2020	★★★	★★	★★	7
**24**	Zou Y 2020	★★★	★★	★★	7
**25**	Zu H 2014	★★★	★	★★	6
**26**	Alshehri A 2020	★★★★	★★	★★	8
**27**	Cho JH 2015	★★★	★★	★★	7
**28**	Men HT 2016	★★★	★★	★	6
**29**	Choi JH 2020	★★★	★★	★★★	8
**30**	Zhang M 2010	★★★★	★★	★★	8
**31**	Chiu CT 2011	★★★★	★	★★	7

**32**	Jiang CG 2011	★★★★	★★	★★	8
**33**	Lee HH 2012	★★★	★	★★	6
**34**	Bu Z 2013	★★★	★	★★	6
**35**	Jiang H 2013	★★★★	★★	★★	8
**36**	Kwon KJ 2014	★★★	★★	★★	7
**37**	Shim JH 2014	★★★★	★	★★	7
**38**	Liu X 2015	★★★★	★★	★★	8
**39**	Hsu JT 2016	★★★★	★★	★★	8
**40**	Kong P 2016	★★★	★★	★	6
**41**	Lu M 2016	★★★★	★★	★★	8
**42**	Tang X 2016	★★★	★★	★★	7
**43**	Wang Z 2016	★★★	★★	★	6
**44**	Chon HJ 2017	★★★★	★★	★★★	9
**45**	Chen J 2018	★★★★	★	★	6
**46**	Lee D 2018	★★★	★	★★	6
**47**	Kao YC 2019	★★★	★★	★★	7
**48**	Ahn H 2020	★★★	★★	★★	7
**49**	Huang KH 2020	★★★	★	★★	6
**50**	Wang JB 2020	★★★★	★★	★★	7
**51**	Dong X 2021	★★★★	★★	★★★	9
**52**	Jin X 2021	★★★	★★	★★	7
**53**	Zhao B 2021	★★★★	★★	★★	8
**54**	Bozkaya Y 2016	★★★	★	★★	6
**55**	Gronnier C 2013	★★★★	★★	★★	8
**56**	Lemoine N 2016	★★★	★★	★★	7
**57**	Riihimäki M 2016	★★★	★★	★	6
**58**	Piessen 2012	★★★	★★	★★	7
**59**	Heger U 2014	★★★	★★	★★	7
**60**	Schmidt T 2014	★★★	★★	★	6
**61**	Voron T 2016	★★★★	★★	★★	8
**62**	Khan N 2020	★★★★	★★★	★★	9
**63**	Shridhar R 2013	★★★★	★★	★★	8
**64**	Taghavi S 2012	★★★★	★	★★	7
**65**	Bamboat ZM 2014	★★★	★★	★★★	8
**66**	Postlewait LM 2015	★★★	★★	★★	7
**67**	Charalampakis N 2016	★★★	★★	★★	7
**68**	Liu K 2017	★★★★	★	★★	7

**69**	Luu C 2017	★★★	★★	★★	7
**70**	Benesch MGK 2020	★★★★	★★	★★★	9
**71**	Tang CT 2020	★★★★	★★	★★	7
**72**	Wei Q 2020	★★★★	★★★	★★	9
**73**	Zhao X 2021	★★★★	★★	★★★	9
**74**	de Aguiar VG 2019	★★★	★	★★	6
