# Supplementary material for: Clinical Features of Gastric Signet Ring Cell Cancer: Results from a Systematic Review and Meta-Analysis
Source: Cancers (Basel). 2023 Oct 28;15(21):5191. doi: 10.3390/cancers15215191 (PMC10647446; doi:10.3390/cancers15215191)
Supplement: Supplementary file 1 [file cancers-15-05191-s001.zip › Table S3.pdf]

Table S3A. General information of the selected studies.

	Author	TNM	T0 SRCC	T0 NSRCC	T1 SRCC	T1 NSRCC	T2 SRCC	T2 NSRCC	T3 SRCC	T3 NSRCC	T4 SRCC	T4 NSRCC	SRCC definition
1	Efared B 2020	7	-	-	-	-	0	21	20	23	2	9	>50% of SRC
2	Lee JH 2010	5	-	-	448	914	-	-	-	-	-	-	>50% of SRC
3	Nam MJ 2010	7	-	-	720	1804	-	-	-	-	-	-	NS
4	Kim HM 2011	7	-	-	419	288	-	-	-	-	-	-	SRCC as part of UDC
5	Tong JH 2011	6	-	-	102	320	-	-	-	-	-	-	>50% of SRC
6	Huh CW 2013	NS	-	-	696	1512	-	-	-	-	-	-	>50% of SRC; mixed SRCC if 10-50%
7	Kim BS 2014	NS	-	-	345	1705	-	-	-	-	-	-	>50% of SRC
8	Guo CG 2015	7	-	-	198	869	-	-	-	-	-	-	>50% of SRC
9	Jin EH 2015	NS	-	-	227	877	-	-	-	-	-	-	according to JGCA
10	Lee SH 2015	NS	-	-	114	582	-	-	-	-	-	-	SRCC as part of UDC
11	Wang Z 2015	NS	-	-	115	219	-	-	-	-	-	-	>50% of SRC; SRCC as part of UDC
12	Hwang CS 2016	NS	-	-	233	317	-	-	-	-	-	-	SRCC as part of UDC
13	Imamura T 2016	7	-	-	190	556	-	-	-	-	-	-	pure SRCC: 100% SRC; mixed SRCC: with other components
14	Kim YH 2016	NS	-	-	927	368	-	-	-	-	-	-	abundant intracellular mucin + compressed displaced nucleus
15	Yoon HJ 2016	NS	-	-	930	2489	-	-	-	-	-	-	SRCC as part of UDC; mixed SRCC if < 50% SRC
16	Bang CS 2017	NS	-	-	89	186	-	-	-	-	-	-	NS
17	Kang Sun H 2017	NS	-	-	91	731	-	-	-	-	-	-	>50% of SRC
18	Lee IS 2017	7	-	-	652	1185	-	-	-	-	-	-	>50% of SRC; mixed SRCC if <50%
19	Horiuchi Y 2018	NS	-	-	129	139	-	-	-	-	-	-	pure SRCC: 100%SRC
20	Kwak DS 2018	NS	-	-	331	206	-	-	-	-	-	-	according to JGCA
21	Nakamura R 2019	NS	-	-	209	117	-	-	-	-	-	-	NS
22	Ryu DG 2019	NS	-	-	233	143	-	-	-	-	-	-	SRCC as part of UDC
23	Zhu ZL 2020	NS	-	-	287	230	-	-	-	-	-	-	according to JGCA; UDC is similar to diffuse
24	Zou Y 2020	NS	-	-	146	177	-	-	-	-	-	-	presence of SRC
25	Zu H 2014	NS	-	-	-	-	18	194	23	431	3	72	NS
26	Alshehri A 2020	8	NS	NS	NS	NS	NS	NS	NS	NS	NS	NS	SRCC as part of UDC
27	Cho JH 2015	NS	NS	NS	NS	NS	NS	NS	NS	NS	NS	NS	NS

**28**	Men HT 2016	NS	NS	NS	NS	NS	NS	NS	NS	NS	NS	NS	NS
**29**	Choi JH 2020	7	NS	NS	NS	NS	NS	NS	NS	NS	NS	NS	NS
**30**	Zhang M 2010	5	-	-	49	89	40	269	98	527	31	336	>50% of SRC
**31**	Chiu CT 2011	NS	-	-	149	430	70	311	268	1070	18	123	>50% of SRC
**32**	Jiang CG 2011	7	-	-	54	215	32	294	46	749	79	846	>50% of SRC
**33**	Lee HH 2012	7	-	-	164	251	32	156	40	255	84	394	>50% of SRC
**34**	Bu Z 2013	5	-	-	T1/2 53	T1/2 26	-	-	T3/4 54	T3/4 48	-	-	>50% of SRC
**35**	Jiang H 2013	5	-	-	68	4	88	15	8	2	124	59	>50% of SRC; SRCC as part of UDC
**36**	Kwon KJ 2014	7	-	-	51	275	9	71	13	122	35	193	NS
**37**	Shim JH 2014	6	-	-	221	1191	34	294	33	298	89	483	>50% of SRC; SRCC as part of UDC
**38**	Liu X 2015	7	NS	NS	NS	NS	NS	NS	NS	NS	NS	NS	>50% of SRC
**39**	Hsu JT 2016	7	-	-	163	103	56	83	19	27	307	712	SRCC as part of UDC
**40**	Kong P 2016	7	-	-	21	211	27	64	29	85	13	30	>50% of SRC
**41**	Lu M 2016	7	NS	NS	NS	NS	NS	NS	NS	NS	NS	NS	>50% of SRC
**42**	Tang X 2016	7	-	-	107	11	14	26	23	52	116	155	>50% of SRC
**43**	Wang Z 2016	7	NS	NS	NS	NS	NS	NS	NS	NS	NS	NS	NS
**44**	Chon HJ 2017	7	-	-	1091	3094	119	771	107	756	329	1400	>50% of SRC
**45**	Chen J 2018	NS	-	-	28	90	8	27	23	50	3	12	>50% of SRC
**46**	Lee D 2018	7	-	-	42	165	26	102	30	159	78	162	>50% of SRC
**47**	Kao YC 2019	8	-	-	185	634	74	402	411	911	85	269	SRCC if >50% SRC in the mucosa irrespective of the deep component
**48**	Ahn H 2020	8	-	-	115	102	15	28	17	73	53	57	>50% of SRC
**49**	Huang KH 2020	8	NS	NS	NS	NS	NS	NS	NS	NS	NS	NS	>50% of SRC
**50**	Wang JB 2020	8	-	-	122	595	35	345	102	634	190	1319	>50% of SRC
**51**	Dong X 2021	8	-	-	62	743	21	487	48	785	123	1870	>50% of SRC; mixed SRCC if <50%
**52**	Jin X 2021	8	NS	NS	NS	NS	NS	NS	NS	NS	NS	NS	according to JGCA; >50% of SRC
**53**	Zhao B 2021	8	-	-	54	225	29	259	73	770	79	402	>50% of SRC; SRCC as part of UDC; SRCC as diffuse
**54**	Bozkaya Y 2016	6	-	-	T1/2 22	T1/2 17	-	-	44	19	76	15	>50% of SRC
**55**	Gronnier C 2013	6	-	-	104	317	-	-	-	-	-	-	>50% of SRC
**56**	Lemoine N 2016	NS	NS	NS	NS	NS	NS	NS	NS	NS	NS	NS	>50% of SRC

**57**	Riihimäki M 2016	NS	-	-	-	-	-	-	-	-	-	-	NS
**58**	Piessen 2012	6	-	-	21	39	25	54	33	47	17	18	>50% of SRC
**59**	Heger U 2014	7	8	25	10	43	12	82	123	284	70	41	>50% of SRC
**60**	Schmidt T 2014	7	-	-	-	-	-	-	-	-	-	-	as described by the WHO
**61**	Voron T 2016	7	-	-	T0/T1 139	T0/T1 212	252	338	361	243	147	107	>50% of SRC and any diffuse-type + SRC
**62**	Khan N 2020	7	6	97	41	636	34	474	84	912	33	170	presence of SRC
**62**	Shridhar R 2013	6	NS	NS	NS	NS	NS	NS	NS	NS	NS	NS	NS
**64**	Taghavi S 2012	6	-	-	510	2098	761	2254	574	1089	501	1082	as described by the WHO
**65**	Bamboat ZM 2014	7	-	-	50	113	18	51	46	116	96	79	>50% of SRC
**66**	Postlewait LM 2015	7	1	5	58	128	29	74	105	142	118	100	as described by the WHO
**67**	Charalampakis N 2016	7	NS	NS	NS	NS	NS	NS	NS	NS	NS	NS	as described by the WHO (Type 1: ≤10%, Type 2: 11-49%, Type 3: ≥50%)
**68**	Liu K 2017	NS	-	-	1099	4727	1442	5456	1012	2511	865	2183	>50% of SRC
**69**	Luu C 2017	NS	-	-	T1/2 22	T1/2 44	-	-	T3/4 17	T3/4 66	-	-	NS
**70**	Benesch MGK 2020	NS	NS	NS	NS	NS	NS	NS	NS	NS	NS	NS	>50% of SRC; mixed SRCC if <50%
**71**	Tang CT 2020	NS	-	-	1182	102	2077	369	1351	199	655	82	abundant intracellular mucin + compressed displaced nucleus
**72**	Wei Q 2020	7	-	-	380	2056	187	921	600	2871	584	1545	>50% of SRC
**73**	Zhao X 2021	7	-	-	811	1263	341	489	916	1173	938	748	NS
**74**	de Aguiar VG 2019	8	NS	NS	NS	NS	NS	NS	NS	NS	NS	NS	>50% of SRC

Abbreviations. JGCA: Japanese Gastric Cancer Association; NS: not specified; NSRCC: Non-Signet Ring Cell Carcinoma; OS: Overall Survival; SRC: Signet Ring Cell; SRCC: Signet Ring Cell Carcinoma; UDC: Undifferentiated Cancer; WHO: World Health Organization.

Table S3B. General information of the selected studies.

	Author	Classification	WHO ed	Japanese ed	Resection	Chemotherapy	Specimen or biopsy	SURVIVAL	HR/RR for SRCC
**1**	Efared B 2020	WHO	4th	-	ev resection	NS	specimen; biopsy	OS	-
**2**	Lee JH 2010	WHO; Nakamura	2nd	-	R0	-	specimen	-	-
**3**	Nam MJ 2010	Lauren; NS; Nakamura	-	-	R0	-	specimen	-	-
**4**	Kim HM 2011	Japanese	-	2nd	R0	-	specimen	-	-
**5**	Tong JH 2011	WHO; Nakamura	3rd	-	R0	-	specimen	OS	-
**6**	Huh CW 2013	WHO	2nd	-	R0	-	specimen	OS	-
**7**	Kim BS 2014	WHO; Japanese	NS	3rd	R0	-	specimen	-	-
**8**	Guo CG 2015	WHO; Japanese; Nakamura	NS	3rd	R0	-	specimen	OS	multiv RR for SRCC vs other
**9**	Jin EH 2015	WHO; Japanese	NS	NS	R0	-	specimen	OS	-
**10**	Lee SH 2015	Japanese	-	2nd	R0	-	specimen	-	-
**11**	Wang Z 2015	WHO; Japanese	3rd	3rd	R0	-	specimen	OS	multiv HR for SRCC vs other
**12**	Hwang CS 2016	Japanese; Lauren; Nakamura	-	3rd	R0	-	specimen	-	-
**13**	Imamura T 2016	NS	-	-	R0	-	specimen	OS	multiv HR for SRCC vs other
**14**	Kim YH 2016	WHO; Japanese; Lauren	4th	NS	R0 (surgery/ESD)	-	specimen	-	-
**15**	Yoon HJ 2016	WHO; Japanese; Lauren	3rd	3rd	R0 (surgery/ESD)	-	specimen	-	-
**16**	Bang CS 2017	NS (Nakamura)	-	-	R0/R+ (ESD)	-	specimen	CRD	-
**17**	Kang Sun H 2017	NS (mix WHO+Japanese)	-	-	R0	-	specimen	-	-
**18**	Lee IS 2017	WHO; Japanese; Lauren	NS	3rd	R0	-	specimen	-	-
**19**	Horiuchi Y 2018	NS (Japanese Nakamura)	-	-	R0 (ESD)	-	specimen	-	-
**20**	Kwak DS 2018	NS (Japanese Nakamura)	-	3rd	R0	-	specimen	-	-
**21**	Nakamura R 2019	NS (Japanese)	-	-	R0	-	specimen	-	-
**22**	Ryu DG 2019	Japanese	-	4th	R0	-	specimen	-	-

**23**	Zhu ZL 2020	Japanese; Lauren; Nakamura	-	3rd	R0	-	specimen	-	-
**24**	Zou Y 2020	NS	-	-	R0	-	specimen	-	-
**25**	Zu H 2014	WHO	2nd	-	R0	NS	specimen	OS	multiv HR for SRCC vs other
**26**	Alshehri A 2020	Japanese	-	NS	R0	ev adjuvant CHT	specimen	OS, RFS	multiv HR for SRCC vs other
**27**	Cho JH 2015	NS	-	-	R0/R+	palliative	not defined	OS	univ+multiv HR for SRCC vs other
**28**	Men HT 2016	NS	-	-	ev resection	IPC/systemic CHT	specimen	OS	univ+multiv HR for SRCC vs other
**29**	Choi JH 2020	NS	-	-	ev resection	palliative	not defined	OS	multiv HR for SRCC vs other
**30**	Zhang M 2010	WHO	4th	-	R0/R+	ev adjuvant CHT	specimen	OS	-
**31**	Chiu CT 2011	WHO	2nd	-	R0	NS	specimen	OS	-
**32**	Jiang CG 2011	WHO	2nd	-	R0/R+	ev adjuvant CHT	specimen	OS	multiv RR for SRCC vs other
**33**	Lee HH 2012	WHO; Japanese; Lauren	2nd	2nd	R0/R+	ev adjuvant CHT	specimen	OS	-
**34**	Bu Z 2013	Japanese	-	1st	R0/R+	NS	specimen	OS	-
**35**	Jiang H 2013	Japanese	-	2nd	R0/R+	NS	specimen	OS	-
**36**	Kwon KJ 2014	NS (Nakamura)	-	-	R0/R+	ev adjuvant CHT	specimen	OS	univ HR for SRCC vs other
**37**	Shim JH 2014	WHO; Japanese mentioned	2nd	2nd	R0	ev adjuvant CHT	specimen	OS	univ+multiv HR for SRCC vs other
**38**	Liu X 2015	WHO	4th	-	R0	NS	specimen	OS	multiv HR for SRCC vs other
**39**	Hsu JT 2016	Japanese	-	3rd	R0/R+	ev adjuvant CHT	specimen	OS	multiv HR for SRCC vs other
**40**	Kong P 2016	WHO	4th	-	R0	ev adjuvant CHT	specimen	OS	univ HR for SRCC vs other
**41**	Lu M 2016	WHO; Lauren	4th	-	R0/R+	ev neoadjuvant/adjuvant/palliative CHT	not defined	OS	univ+multiv HR for SRCC vs other
**42**	Tang X 2016	WHO; Lauren	4th	-	R0	ev adjuvant CHT	specimen	OS	-
**43**	Wang Z 2016	NS	NS	NS	R0	ev adjuvant CHT	specimen	OS	univ HR for SRCC vs other
**44**	Chon HJ 2017	WHO; Lauren	4th	-	R0	NS	specimen	OS, RFS	multiv HR for SRCC vs other
**45**	Chen J 2018	WHO	2nd	-	R0	NS	specimen	-	-
**46**	Lee D 2018	NS	-	-	R0	ev adjuvant CHT	specimen	DFS, CSS	-

**47**	Kao YC 2019	NS	-	-	R0	NS	specimen	OS, DFS	multiv HR for SRCC vs other
**48**	Ahn H 2020	WHO	NS	-	R0/R+	ev adjuvant CHT	specimen	OS	univ+multiv HR for SRCC vs other
**49**	Huang KH 2020	WHO	2nd	-	R0	ev adjuvant CHT	specimen	OS	univ RR for SRCC vs other
**50**	Wang JB 2020	WHO	4th	-	R0	ev adjuvant CHT	specimen	OS	-
**51**	Dong X 2021	WHO	4th	-	R0	ev adjuvant CHT	specimen	OS	univ+multiv HR for SRCC vs other
**52**	Jin X 2021	Japanese; Nakamura	-	3rd	R0	-	specimen	-	-
**53**	Zhao B 2021	WHO; Japanese	NS	2nd	R0	ev adjuvant CHT	specimen	DFS	-
**54**	Bozkaya Y 2016	WHO	2nd	-	R0/R+	ev adjuvant CHT	specimen	OS	-
**55**	Gronnier C 2013	WHO	2nd	-	R0/R+	ev adjuvant CHT	specimen	OS	multiv HR for SRCC vs other
**56**	Lemoine N 2016	WHO	2nd	-	ev resection	palliative	not defined	OS, PFS	univ+multiv HR for SRCC vs other
**57**	Riihimäki M 2016	WHO; Lauren	NS	-	NS	NS	not defined	OS	multiv HR for SRCC vs other
**58**	Piessen 2012	WHO	2nd	-	R0/R+	NS	biopsy	OS	multiv HR for SRCC vs other
**59**	Heger U 2014	WHO; Lauren	NS	-	ev resection R0/R+	neoadjuvant CHT	not defined	OS	multiv RR for SRCC vs other
**60**	Schmidt T 2014	WHO	NS	-	R0/R+	neoadjuvant	specimen	OS	multiv RR for SRCC vs other
**61**	Voron T 2016	WHO; Lauren	2nd	-	R0/R+	ev neoadjuvant/adjuvant CHT	specimen	OS	multiv HR for SRCC vs other
**62**	Khan N 2020	NS (WHO)	-	-	R0/R+	ev neoadjuvant/adjuvant CHT	specimen	OS	univ HR for SRCC vs other
**63**	Shridhar R 2013	NS	-	-	ev resection	palliative	not defined	OS	multiv HR for SRCC vs other
**64**	Taghavi S 2012	WHO	2nd	-	NS	NS	not defined	OS	univ+multiv HR for SRCC vs other
**65**	Bamboat ZM 2014	WHO; Lauren	1st	-	R0	ev adjuvant CHT	specimen	OS	-
**66**	Postlewait LM 2015	WHO	4th	-	R0/R+	ev neoadjuvant/adjuvant CHT	specimen	OS, RFS	multiv HR for SRCC vs other
**67**	Charalampakis N 2016	WHO	4th	-	R0/R+	neoadjuvant CRT	specimen	OS, RFS	univ+multiv HR for SRCC vs other
**68**	Liu K 2017	WHO	4th	-	NS	NS	not defined	OS	-
**69**	Luu C 2017	Lauren	-	-	R0/R+	ev neoadjuvant/adjuvant CHT	specimen	OS	multiv HR for SRCC vs other
**70**	Benesch MGK 2020	WHO	5th	-	ev resection	ev CHT/RT	not defined	OS	univ+multiv HR for SRCC vs other
**71**	Tang CT 2020	WHO	5th	-	resection	ev adjuvant CHT	specimen	OS, CSS	multiv HR for SRCC vs other

**72**	Wei Q 2020	WHO	4th	-	NS	NS	NS	OS	-
**72**	Zhao X 2021	WHO	4th	-	ev resection	NS	NS	OS, CSS	multiv HR for SRCC vs other
**74**	de Aguiar VG 2019	WHO; Lauren	4th	-	ev resection	ev adjuvant CHT	not defined	OS	multiv HR for SRCC vs other

Abbreviations. CHT: Chemotherapy; CRD: Cancer Related Death; CSS: Cancer Specific Survival; DFS: Disease Free Survival; ESD: Endoscopic Submucosal Dissection; ev: eventual; IPC: Intraperitoneal Chemotherapy; JGCA: Japanese Gastric Cancer Association; MDA: Moderately Differentiated Adenocarcinoma; multiv: multivariate; NS: not specified; OS: Overall Survival; PDA: Poorly Differentiated Adenocarcinoma; PFS: Progression Free Survival; RFS: Relapse Free Survival; R0: radical resection; R+: non-radical resection; RT: Radiotherapy; SRC: Signet Ring Cell; SRCC: Signet Ring Cell Carcinoma; UDC: Undifferentiated Cancer; univ: univariate; WDA: Well Differentiated Adenocarcinoma; WHO: World Health Organization; WMDA: Well/Moderately Differentiated Adenocarcinoma.
