# Supplementary figures and images for: Clinical Features of Gastric Signet Ring Cell Cancer: Results from a Systematic Review and Meta-Analysis
Source: Cancers (Basel). 2023 Oct 28;15(21):5191. doi: 10.3390/cancers15215191 (PMC10647446; doi:10.3390/cancers15215191)

**Figure S4** Funnel plot and Egger's Test

**Figure S4A** All stage GC Survival

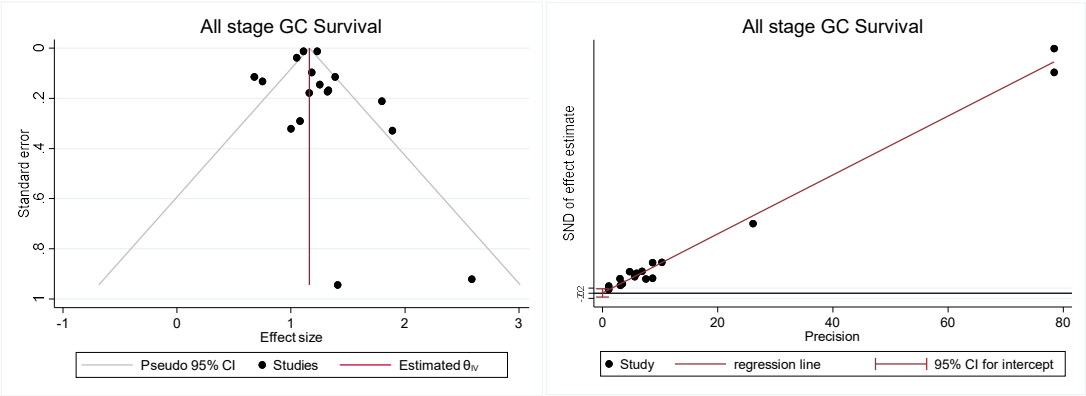

**Figure S4B** Early GC Survival

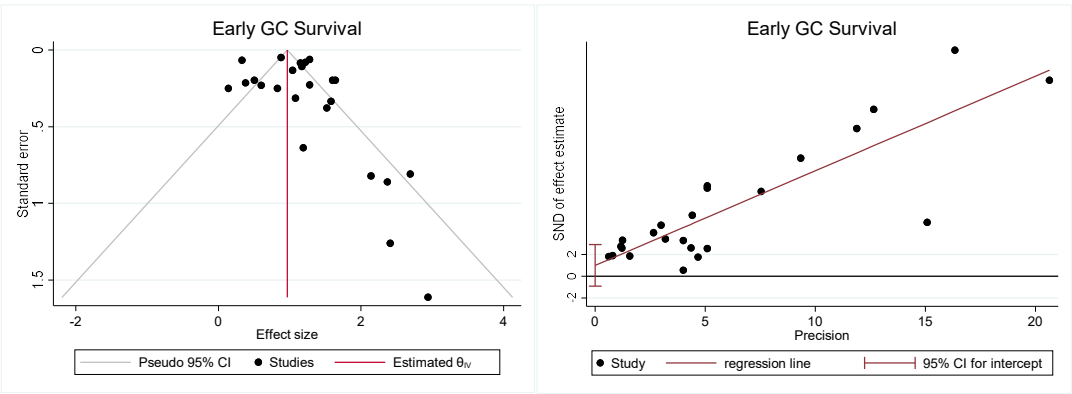

Supplement: Supplementary file 1 [file cancers-15-05191-s001.zip › Figure S4.pdf]
